# Expression of human chorionic gonadotropin beta in gastric carcinoma: A retrospective immunohistochemical study

**DOI:** 10.4103/0971-5851.64254

**Published:** 2009

**Authors:** Kanchan M. Murhekar, Jayaram N. Anuratha, Urmila Majhi, Thangarajan Rajkumar

**Affiliations:** *Departments of Pathology, Cancer Institute (WIA), Adyar, Chennai, India*; 1 *Departments of Immunology, Cancer Institute (WIA), Adyar, Chennai, India*; 2 *Departments of Molecular Oncology, Cancer Institute (WIA), Adyar, Chennai, India*

**Keywords:** βHCG, gastric carcinoma, immunohistochemistry, prognosis

## Abstract

**Context:**

Beta Human Chorionic Gonadotropin (βHCG), a marker of the trophoblastic neoplasm, is also secreted by non-trophoblastic neoplasms including gastric carcinomas. Its role in disease progression remains unclear.

**Aim:**

To investigate the incidence of βHCG positivity in gastric carcinomas and correlate its presence with the biological behavior of the tumor.

**Setting and Design:**

A hospital-based, immunohistochemical study.

**Materials and Methods:**

One hundred and fifty formalin-fixed, paraffin-embedded tissue specimens from histopathologically confirmed cases of gastric carcinoma were immunostained using an indigenously developed antibody against βHCG. Tumors with diffuse reactivity to βHCG were considered as positive. Those with occasional, focal or no reactivity to βHCG were considered as negative.

**Statistical Analysis:**

Differences in βHCG staining were compared according to the histological grade and surgical stage using the χ^2^ test. Using the Cox proportional hazards model, the time till the onset of development of an adverse outcome after surgery (defined as death, local or distant metastasis) was compared between the bHCG positive and negative tumors.

**Results:**

Twenty-eight (18.7%) of the 150 specimens were βHCG positive. No association was found between the histological grade (*P*=0.49) and the surgical stage (*P*=0.19) with βHCG positivity. The median disease-free survival after surgery was not different among βHCG positive and negative tumors. Risk of an adverse outcome after surgery was significantly associated with the stage of the tumor (Hazard ratio=2.9, 95% confidence interval: 1.1–7.4). No association was observed with grade or βHCG positivity.

**Conclusion:**

βHCG immunoreactivity was observed in about one-fifth of the gastric cancers. βHCG reactivity, however, played no role in the biological behavior.

## INTRODUCTION

Human Chorionic Gonadotropin (HCG) is a glycoprotein hormone that biochemically consists of two polypeptide subunits (alpha and beta chains), with attached carbohydrate side chains.[1,2] The hormone is normally produced by a placental syncytiotrophoblast and also by neoplastic cells in the tumors of trophoblastic origin. However, it is wellestablished that production of HCG is not restricted to these tumors alone and this hormone is secreted by several non-trophoblastic neoplasms including colorectal, breast, gastric, bronchogenic, and bladder cancers.[3–6] Several studies have shown that HCG secreting neoplasms have a more aggressive behavior.[4,7]

A large proportion of gastrointestinal carcinomas have been shown to secrete HCG.[8] Few studies have reported Beta Human Chorionic Gonadotropin (βHCG) positivity in gastric carcinomas by immunohistochemical techniques.[7,9] However, its role in disease progression is not clear, with some studies showing a definite association of βHCG positive tumors with poor prognosis,[7] and some showing no such association.[9] In India, stomach cancer is the second most common cancer. Annually, more than 1000 cases of gastric carcinoma are seen in our institute, which is one of the largest oncology centers in India. However, very few undergo surgery with a curative intent, as the vast majority is too advanced for any curative approach. As no data is available on the βHCG reactivity among the gastric tumors from India, we conducted a study to investigate the incidence of βHCG positivity in gastric carcinomas, using an indigenously developed antibody against βHCG[10] and correlate its presence with the biological behavior of the tumor. Kabeer *et al*. had also reported membrane staining in some of the cancers that they had tested. This finding potentially offers the option of targeted therapy using a monoclonal antibody.[10]

## MATERIALS AND METHODS

### Tissue samples

Formalin-fixed and paraffin-embedded tissue specimens from 150 clinically diagnosed and histopathologically confirmed cases of gastric carcinoma admitted in our institute for surgery during 2003–2007 were included in the study. All these patients underwent distal / subtotal gastrectomy with nodal dissection and none had received chemotherapy prior to surgery.

### Reagents

We used an indigenously developed chimeric, recombinant antibody, engineered from a mouse monoclonal, raised against βHCG (primary antibody). The antibody was a recombinant protein having human IgG1 as a constant heavy chain and human kappa as a constant light chain, fused with variable chains of mouse monoclonal antibody. The antibody is specific to HCG and does not recognize human TSH and FSH, both sharing the same alpha chain with HCG.[10] We used Horse Radish peroxidase (HRP)-conjugated goat anti-human IgG (Jackson Immunoresearch) as a secondary antibody. This indigenously produced antibody was found to be immunoreactive in both early and advanced stages of different types of cancers (Dr. Prem Chopra, Sir Ganga Ram Hospital, Personal communication)

### Controls

We used sections of formalin-fixed, paraffin-embedded, normal human placenta as the control.

### Immuno-staining procedure

We used a two-step peroxidase anti-peroxidase (PAP) procedure for immunostaining of the sections. Four-micron sections of a formalin-fixed tumor tissue were prepared. After de-paraffinization, the sections were treated with 3% hydrogen peroxide for 30 minutes at room temperature, to eliminate the endogenous peroxidase activity. No antigen retrieval was done. After washing in phosphate buffered saline (PBS), all specimens were incubated overnight with a primary antibody in 1:40 dilution. After washing thoroughly with tap water, sections were incubated with a secondary antibody in 1:400 dilution for 40 minutes at room temperature. 2-4 diamino benzidine (DAB, Sigma) was used as a substrate to develop the signal, and sections were counter-stained with hematoxylin, and dehydrated and mounted in DPX mounting medium. This immunostaining procedure was also followed for the formalin-fixed, paraffin-embedded, normal human placenta, which served as the positive control. The negative control sections of the normal human placenta were subjected to the same procedures, omitting the primary antibody. The stained slides were examined microscopically for the presence of stained tumor cells.

The βHCG reactivity in gastric tumors ranged from occasional (only few isolated reactive cells) to focal (clusters of positive cells in some areas of the tumor, but the other regions were negative) to diffuse reactivity (isolated and / or cluster of positive cells were found throughout most areas of the tumor). However, considering the potential biological relevance, we considered tumors with diffuse reactivity to βHCG as positive and those with occasional or focal βHCG reactive cells as negative.

### Statistical analysis

We compared the histopathological grade, surgical stage, and biological behavior of the tumor among βHCG positive and negative tumors. The different grades and stages were compared for differences in staining using the χ^2^ test. We assessed the biological behavior of the tumor by comparing the time till the onset of development of an adverse outcome after surgery among the βHCG positive and negative tumors using the Cox proportional hazards regression model. Death of the patient or development of local or distant metastasis after surgery was considered as an adverse outcome. We calculated the hazards ratio and 95% confidence limits for βHCG reactive tumors as well other covariates (stage and grade of the tumor). Patients with stage IV tumors were excluded from this analysis. We analyzed the data using the Epi-Info software.

## RESULTS

**Figure 1:**
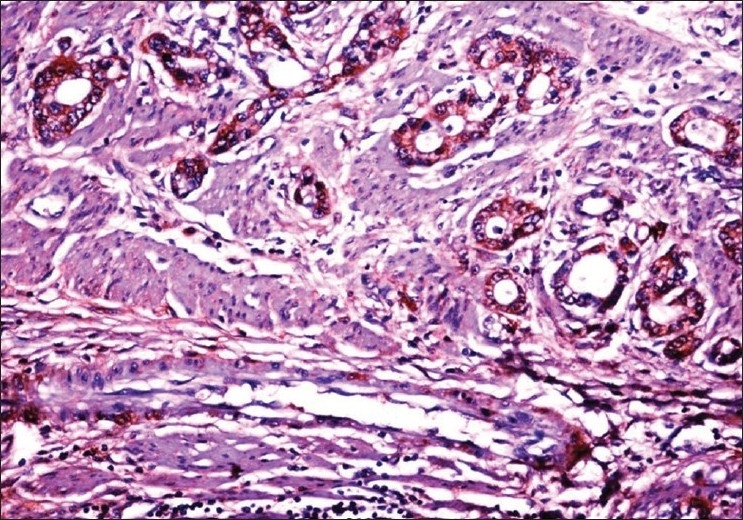
Grade-2 adenocarcinoma of the stomach showing βHCG positive cells (40×)

**Figure 2:**
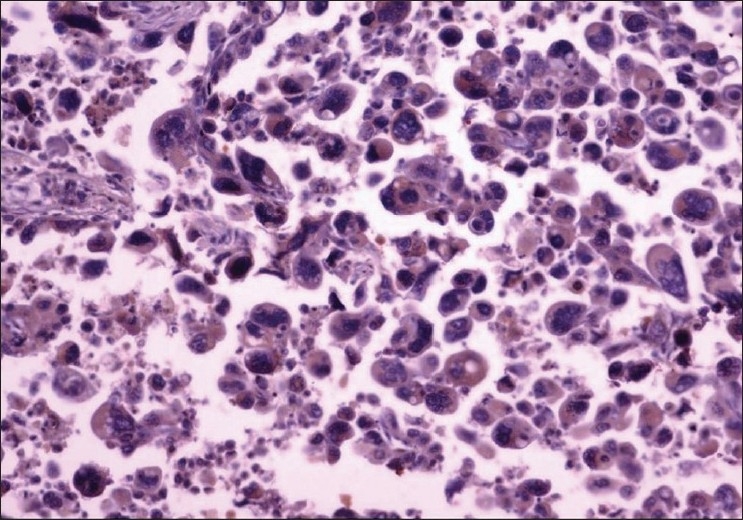
Grade-3 adenocarcinoma of stomach showing βHCG positive cells (40×)

Tissue samples of gastric carcinoma from 150 patients were included in the study. The median age of the patients was 56 years (range: 25–75 years) and 113 (75%) were males. Histologically, 113 (75%) tumors were of grade-3 (poorly differentiated), whereas, 37 (25%) were of grade-2. According to the TNM classification, 18 (12%) were of stage-1, 31 (21%) were of stage-2, 88 (59%) were of stage-3, and 13 (9%) were of stage-4. Of the 150 tissue samples, 28 (18.7%) showed βHCG positivity. The immunoreactivity to βHCG was evidenced by a diffuse brown staining in the cytoplasm of the tumor cells [Figures 1 and 2]; we did not observe any membrane staining in our cases.

No association was found between the histological grade (χ^2^ = 0.47, *P* = 0.49) or the surgical stage (χ^2^  = 4.8, *P* = 0.19) with the βHCG positivity [Table 1]. The median disease-free survival among the βHCG positive and negative tumors after surgery was 537 (range: 84–2162) and 584 (range: 39–2078) days, respectively. Of the three independent variables included in the Cox proportional hazards model, the stage of the tumor was the only variable found to be significantly associated with an increased risk of adverse outcome after surgery (Hazard ratio=2.9, 95% confidence interval : 1.1–7.4). The risk of an adverse outcome was higher among βHCG reactive tumors (Hazard ratio=1.5, 95% confidence interval: 0.9–2.5), although the increase was not statistically significant [Table 2].

**Table 1:** βHCG immunoreactivity of gastric tumors according to histological grade and TNM stage

Histological grade and stage	Number	No. (%) positive for βHCG	*P*
Grade			
2	37	5 (13.5)	0.49
3	113	23 (20.4)	
Stage			
1	18	0 (0)	0.19
2	31	6 (19.4)	
4	13	3 (23.1)	

**Table 2:** Hazard ratio and their 95% confidence interval associated with the adverse outcome after surgery

Variables	Hazard ratio	95% CI
βHCG positivity	1.5	0.9 – 2.5
Tumor stage (2 vs. 1)	1.0	0.3 – 3.0
Tumor stage (3 vs. 1)	2.9	1.1 – 7.4
Tumor grade	1.0	0.6 – 1.8

## DISCUSSION

The present study was conducted to find the incidence of βHCG positivity in gastric carcinomas and its correlation with the biological behavior of the tumor in terms of pathological grade, surgical stage, and prognosis, measured as a disease-free survival after surgery. Our study revealed that diffuse βHCG immunoreactive tumor cells were present in about one-fifth of the gastric cancers. No association, however, was observed between the biological behavior of the cancer and the immunoreactivity to βHCG. βHCG immunoreactivity in gastric tumors observed in our study was lower than the positivity of 34-53% reported in other studies. In these studies, the tumors with occasional and focal βHCG reactivity were considered as positive.[9] In our study, however, we considered tumors with diffuse reactivity to βHCG as positives and those with occasional or focal βHCG reactive cells as negative, as our immunohistochemical scoring was based on the potential biological relevance. The low positivity in our study could be on account of this reason.

In our study, 19–22% of the gastric tumors of stage 2 and above were positive for βHCG, whereas, none of the stage-1 tumors were positive for βHCG. The positivity was also higher among grade-3 tumors compared to grade-2 tumors. Similar findings were also reported in other studies.[9] Although the exact mechanism for βHCG production in non-trophoblastic tumors is not known, it is suggested that this might be an event in the course of de-differentiation.[9] The higher proportion of βHCG reactivity in patients having poorly differentiated tumors and tumors of advanced stage could be on account of the theory of de-differentiation.

The role of βHCG reactivity in the prognosis of gastric tumors is unclear. Some studies have reported that patients with gastric cancer and high levels of HCG in the serum or a high density of HCG positive cells in the tumor tissue have a poor prognosis.[7] Wittekind *et al*. also reported that HCG positive tumors have a shorter survival time, although they do not have any correlation of HCG positivity with the stage and grade of the tumor.[8] On the other hand, Yakeishi *et al*. found that βHCG has no prognostic significance.[9] In our study, we found that the overall survival after surgery is not different among βHCG positive and negative tumors, although the positive tumors have a nonsignificant increased risk of adverse outcome after surgery.

In conclusion, the findings of our study indicated that βHCG was produced by about one-fifth of the gastric carcinomas. The production of βHCG, however, was not found to be associated with the pathological grade and surgical stage. The median disease-free survival after surgery among βHCG positive tumors was not different from βHCG negative tumors. The risk of an adverse outcome after surgery was also similar among βHCG positive and negative tumors, indicating that βHCG positivity has no prognostic significance.
